# Supplementary material for: Nuclear size and physical properties of the nucleoplasm are determined by colloid osmotic pressure at the nuclear envelope
Source: bioRxiv. 2026 Jul 23:2026.07.21.739918. Preprint. [Version 1] doi: 10.64898/2026.07.21.739918 (PMC13419497; doi:10.64898/2026.07.21.739918)
Supplement: 1 [file NIHPP2026.07.21.739918v1-supplement-1.pdf]

bioRxiv preprint doi: https://doi.org/10.64898/2026.07.21.739918; this version posted July 23, 2026. The copyright holder for this preprint (which was not certified by peer review) is the author/funder, who has granted bioRxiv a license to display the preprint in perpetuity. It is made available under a CC-BY 4.0 International license.

Supplementary Material

Table S1

S. pombe strains Designation	Source or reference	Identifiers	Additional information
Ish1-GFP, pTOW-M-empty	This work	JL378 / FC	*h+ ish1-GFP:kanMX pTOW-M-ptdh1-empty ade6-M216 leu1-32 ura4-D18 his3-D1*
Ish1-GFP, pTOW-M-mCherry-GST-NLS	This work	JL370 / FC	*h+ ish1-GFP:kanMX pTOW-M-ptdh1-mCherry-GST-NLS ade6-M216 leu1-32 ura4-D18 his3-D1*
Ish1-GFP, pTOW-M-mCherry-GST-linker-MAPKK NES	This work	JL373 / FC	*h+ ish1-GFP:kanMX pTOW-M-ptdh1-mCherry-GST-linker-MAPKK NES ade6-M216 leu1-32 ura4-D18 his3-D1*
Ish1-GFP, pTOW-M-mCherry	This work	JL401 / FC	*h+ ish1-GFP:kanMX pTOW-M-ptdh1-mCherry ade6-M216 leu1-32 ura4-D18 his3-D1*
Ish1-GFP, mCherry-Psy1	Chang Lab collection	JL30 / FC3318	*h- ade6 <<mCherry-psy1 ish1-GFP:kanMX ura4-D18*
Ish1-GFP, hta1-mCherry	This work	JL396 / FC	*h+ hta1-mCherry:KanMX ish1-GFP:kanMX ade6-M216 leu1-32 ura4-D18 his3-D1*
Ish1-GFP	This work	JL308 / FC	*h- ish1-GFP:Nat leu1-32*
Ish1-mScarlet, pREp ptdh1-sfGFP-GST-NLS	This work	JL320 / FC	*h- pREp ptdh1-sfGFP-GST-NLS ish1-mScarlet:Hph leu1-32*
Ish1-GFP, 3X-mCherry-GST-NLS	This work	JL356 / FC	*h- pREp ptdh1-mCherry-(BlackCherry)2-NLS ish1:GFP:Nat leu1-32*
Ish1-GFP, pREp ptdh1-mCherry-GST-NLS	This work	JL318 / FC	*h- pREp ptdh1-mCherry-GST-NLS ish1:GFP:Nat leu1-32*
Ish1-GFP, pREp ptdh1-mCherry-GST-NES	This work	JL358 / FC	*h- pREp ptdh1-mCherry-GST-linker-MAPKK NES ish1:GFP:Nat leu1-32*
Ish1-GFP, Eno1-mCherry	This work	JL408 / FC	*h+ ish1-GFP:kanMX eno1-mCherry:Hph ade6-M216 leu1-32 ura4-D18 his3-D1*
Ish1-GFP, Tdh1-mCherry	This work	JL406 / FC	*h+ ish1-GFP:kanMX tdh1-mCherry:Hph ade6-M216 leu1-32 ura4-D18 his3-D1*
Ish1-GFP, 1X*mCherry-GST-NLS*	This work	JL330 / FC	*h- Δura4::ptdh1-mCherry-GST-NLS ish1:GFP:Nat leu1-32*
NucGEMs, pTOW-M-empty	This work	JL383 / FC	*h- Δura4::pRga3:NLS-Pfv-GS-Sapphire pTOW-M-ptdh1-empty ade6-M216 leu1-32 ura4-D18 his3-D1*

bioRxiv preprint doi: https://doi.org/10.64898/2026.07.21.739918; this version posted July 23, 2026. The copyright holder for this preprint (which was not certified by peer review) is the author/funder, who has granted bioRxiv a license to display the preprint in perpetuity. It is made available under a CC-BY 4.0 International license.

NucGEMs, pTOW-M-mCherry-GST-NLS	This work	JL392 / FC	*h- Δura4::pRga3:NLS-Pfv-GS-Sapphire pTOW-M-ptdh1-mCherry-GST-NLS ade6-M216 leu1-32 ura4-D18 his3-D1*
NucGEMs, pTOW-M-mCherry-GST-linker-MAPKK NES	This work	JL393 / FC	*h- Δura4::pRga3:NLS-Pfv-GS-Sapphire pTOW-M-ptdh1-mCherry-GST-linker-MAPKK NES ade6-M216 leu1-32 ura4-D18 his3-D1*
NucGEMs, pTOW-M-mCherry	This work	JL399 / FC	*h- Δura4::pRga3:NLS-Pfv-GS-Sapphire pTOW-M-ptdh1-mCherry ade6-M216 leu1-32 ura4-D18 his3-D1*
CytGEMs, pTOW-M-empty	This work	JL388 / FC	*h- Δura4::pHis3:NLS-Pfv-GS-Sapphire pTOW-M-ptdh1-empty ade6-M216 leu1-32 ura4-D18 his3-D1*
CytGEMs, pTOW-M-mCherry-GST-NLS	This work	JL394 / FC	*h- Δura4::pHis3:NLS-Pfv-GS-Sapphire pTOW-M-ptdh1-mCherry-GST-NLS ade6-M216 leu1-32 ura4-D18 his3-D1*
CytGEMs, pTOW-M-mCherry-GST-linker-MAPKK NES	This work	JL395 / FC	*h- Δura4::pHis3:NLS-Pfv-GS-Sapphire pTOW-M-ptdh1-mCherry-GST-linker-MAPKK NES ade6-M216 leu1-32 ura4-D18 his3-D1*
CytGEMs, pTOW-M-mCherry	This work	JL400 / FC	*h- Δura4::pHis3:NLS-Pfv-GS-Sapphire pTOW-M-ptdh1-mCherry ade6-M216 leu1-32 ura4-D18 his3-D1*
Swi6-GFP, pTOW-M-empty	This work	JL419 / FC	*h- swi6-sfGFP:KanMX ish1-CFP:kanMX pTOW-M-ptdh1-empty ade6-M216 leu1-32 ura4-D18 his-*
Swi6-GFP, pTOW-M-mCherry-GST-NLS	This work	JL417 / FC	*h- swi6-sfGFP:KanMX ish1-CFP:kanMX pTOW-M-ptdh1-mCherry-GST-NLS ade6-M216 leu1-32 ura4-D18 his-*
Swi6-GFP, pTOW-M-mCherry-GST-linker-MAPKK NES	This work	JL418 / FC	*h- swi6-sfGFP:KanMX ish1-CFP:kanMX pTOW-M-ptdh1-mCherry-GST-linker-MAPKK NES ade6-M216 leu1-32 ura4-D18 his-*
Swi6-GFP, pTOW-M-mCherry	This work	JL420 / FC	*h- swi6-sfGFP:KanMX ish1-CFP:kanMX pTOW-M-ptdh1-mCherry ade6-M216 leu1-32 ura4-D18 his-*
10xSUMO-6xSIM-GFP, pTOW-M-empty	This work	JL434 / FC	*h- pact1-NLS-sfGFP-10xSumo-6xSIM<<ade6 ish1-CFP:kanMX*

bioRxiv preprint doi: https://doi.org/10.64898/2026.07.21.739918; this version posted July 23, 2026. The copyright holder for this preprint (which was not certified by peer review) is the author/funder, who has granted bioRxiv a license to display the preprint in perpetuity. It is made available under a CC-BY 4.0 International license.

			pTOW-M-ptdh1-empty ade6-M216 leu1-32 ura4-D18 his-
10xSUMO-6xSIM-GFP, pTOW-M-mCherry-GST-NLS	This work	JL432 / FC	*h- pact1-NLS-sfGFP-10xSumo-6xSIM<<ade6 ish1-CFP:kanMX pTOW-M-ptdh1-mCherry-GST-NLS ade6-M216 leu1-32 ura4-D18 his-*
10xSUMO-6xSIM-GFP, pTOW-M-mCherry-GST-linker-MAPKK NES	This work	JL433 / FC	*h- pact1-NLS-sfGFP-10xSumo-6xSIM<<ade6 ish1-CFP:kanMX pTOW-M-ptdh1-mCherry-GST-linker-MAPKK NES ade6-M216 leu1-32 ura4-D18 his-*
10xSUMO-6xSIM-GFP, pTOW-M-mCherry	This work	JL435 / FC	*h- pact1-NLS-sfGFP-10xSumo-6xSIM<<ade6 ish1-CFP:kanMX pTOW-M-ptdh1-mCherry ade6-M216 leu1-32 ura4-D18 his-*
2x(10xSUMO-6xSIM-GFP), pTOW-M-empty	This work	JL447 / FC	*h- pact1-NLS-sfGFP-10xSumo-6xSIM<<his5 pact1-NLS-sfGFP-10xSumo-6xSIM<<ade6 ish1-CFP:kanMX pTOW-M-ptdh1-empty ade6-M216 leu1-32 ura4-D18 his-*
2x(10xSUMO-6xSIM-GFP), pTOW-M-mCherry-GST-NLS	This work	JL445 / FC	*h- pact1-NLS-sfGFP-10xSumo-6xSIM<<his5 pact1-NLS-sfGFP-10xSumo-6xSIM<<ade6 ish1-CFP:kanMX pTOW-M-ptdh1-mCherry-GST-NLS ade6-M216 leu1-32 ura4-D18 his-*
2x(10xSUMO-6xSIM-GFP), pTOW-M-mCherry-GST-linker-MAPKK NES	This work	JL446 / FC	*h- pact1-NLS-sfGFP-10xSumo-6xSIM<<his5 pact1-NLS-sfGFP-10xSumo-6xSIM<<ade6 ish1-CFP:kanMX pTOW-M-ptdh1-mCherry-GST-linker-MAPKK NES ade6-M216 leu1-32 ura4-D18 his-*
2x(10xSUMO-6xSIM-GFP), pTOW-M-mCherry	This work	JL448 / FC	*h- pact1-NLS-sfGFP-10xSumo-6xSIM<<his5 pact1-NLS-sfGFP-10xSumo-6xSIM<<ade6 ish1-CFP:kanMX pTOW-M-ptdh1-mCherry ade6-M216 leu1-32 ura4-D18 his-*

bioRxiv preprint doi: https://doi.org/10.64898/2026.07.21.739918; this version posted July 23, 2026. The copyright holder for this preprint (which was not certified by peer review) is the author/funder, who has granted bioRxiv a license to display the preprint in perpetuity. It is made available under a CC-BY 4.0 International license.

Supplemental figures

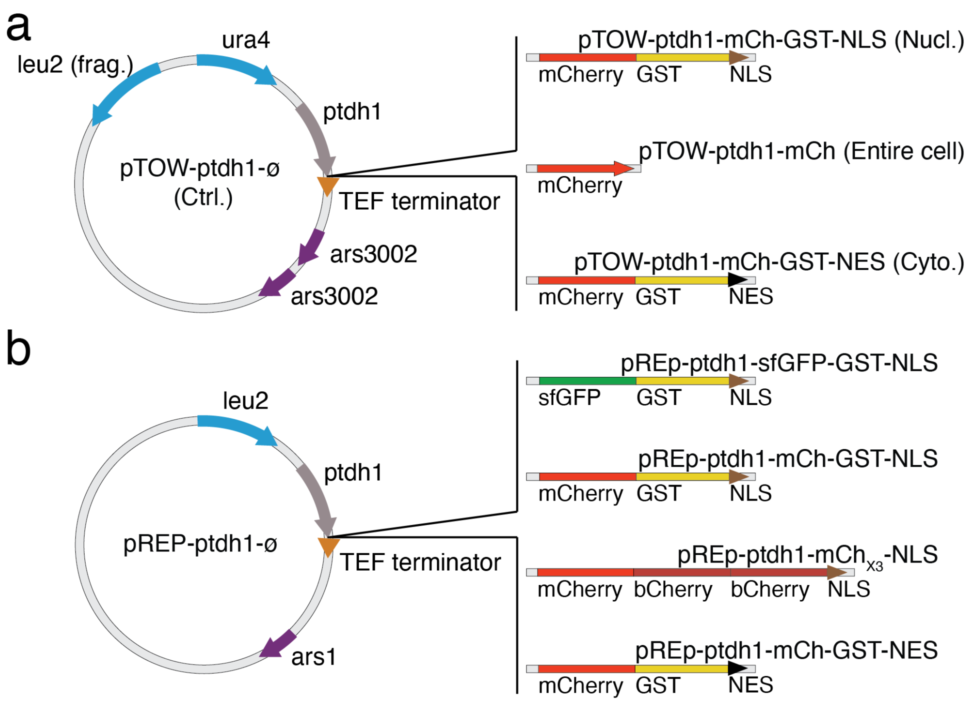

Supplementary Fig. 1 | Schematic of plasmid constructs. a, Schematic of the multi-copy plasmid construction based on the pTOW-ptdh1 backbone expressing an mCherry-tagged protein. **b**, Examples of different plasmids leading to various protein combination constructs where the promoter, fluorophore and GST were swapped compared to the pTOW backbone in **a**.

bioRxiv preprint doi: https://doi.org/10.64898/2026.07.21.739918; this version posted July 23, 2026. The copyright holder for this preprint (which was not certified by peer review) is the author/funder, who has granted bioRxiv a license to display the preprint in perpetuity. It is made available under a CC-BY 4.0 International license.

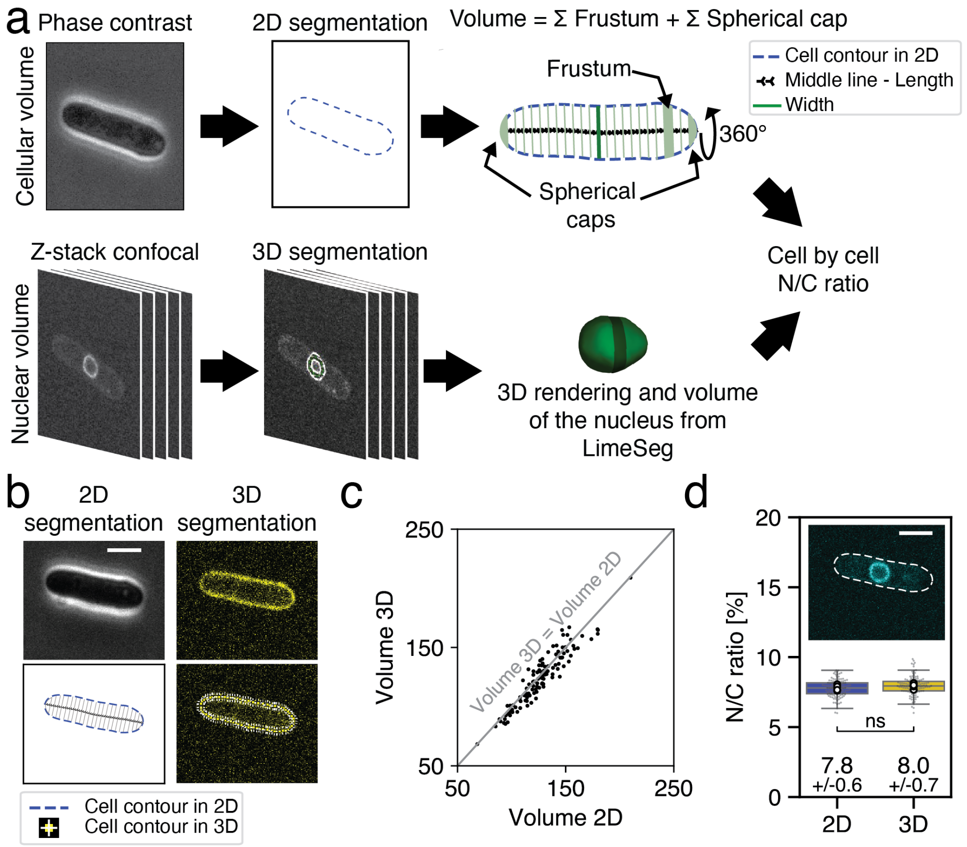

Supplementary Fig. 2 | Cellular and nuclear volume measurement pipeline. **a,** Schematic illustrating the methods for measurement of the N/C ratio for individual cells. A phase-contrast image of the mid-plane is used to segment the cell in 2D. Principal component analysis of the cell contour defines the major axis and midline, along which a stack of frustums matching the cell outline is constructed. Skeletonization is used to segment the cell into frustums of equal width, with spherical caps at each cell tip. Assuming rotational symmetry around the midline, the total cell volume is calculated (top). Bottom, nuclear volume determination from a 3D confocal z-stack. The nuclear envelope is segmented using the LimeSeg plugin, yielding a 3D rendering and nuclear volume. **b,** Representative mid-plane phase-contrast and confocal images of the same cell expressing a plasma membrane marker (yellow), used to compare cell volume measurements obtained from 2D segmentation with those obtained from full 3D segmentation using LimeSeg. Scale bar, 5 µm. **c,** Comparison of cellular volumes calculated using the 2D segmentation pipeline and 3D LimeSeg-based segmentation for a population of fission yeast cells (N = 118), showing good agreement between methods. **d,** Comparison of N/C ratio measurements for the same cell population as in (c) obtained using combined 2D cell volume and 3D nuclear volume measurements versus fully 3D segmentation for both cell and nucleus, demonstrating consistent results across methods. Replicate-level paired permutation test comparing N/C ratios between 2D and 3D conditions. Each replicate's mean was used to account for replicate-to-replicate variability, indicating no significant difference. Mean N/C ratio values and standard deviation.

bioRxiv preprint doi: https://doi.org/10.64898/2026.07.21.739918; this version posted July 23, 2026. The copyright holder for this preprint (which was not certified by peer review) is the author/funder, who has granted bioRxiv a license to display the preprint in perpetuity. It is made available under a CC-BY 4.0 International license.

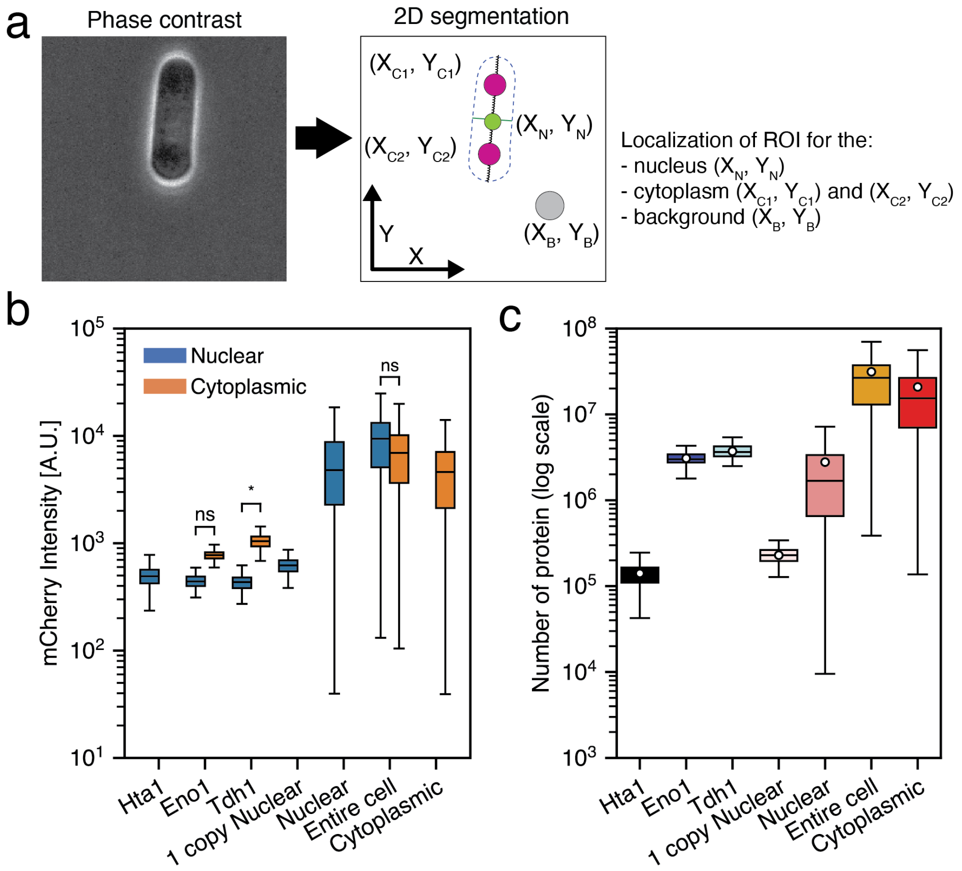

Supplementary Fig. 3 | Proteins intensity measurements and quantification analysis. **a,** Representative phase-contrast image of fission yeast cell used for 2D-sgmentation (left), showing the region of interests (ROIs) automatically selected after segmentation to measure fluorescence intensities in the nucleus, cytoplasm and the background (right). Two cytoplasmic ROIs were defined to account for potential asymmetric protein distribution between the tips. **b,** Quantification of mCherry fluorescence intensity for each condition (**see Fig. 1a**), showing the distribution of the signal between the nucleus (blue) and the cytoplasm (gold). When indicated, nuclear and cytoplasmic intensities from the same cells were compared using a two-sided Wilcoxon signed-rank test. Note that the Entire Cell strain shows no significant difference between the nuclear and cytoplasmic mCherry fluorescence intensities (N≥82 cells per conditions) **c,** Quantification of total mCherry protein levels per cells for each condition.

bioRxiv preprint doi: https://doi.org/10.64898/2026.07.21.739918; this version posted July 23, 2026. The copyright holder for this preprint (which was not certified by peer review) is the author/funder, who has granted bioRxiv a license to display the preprint in perpetuity. It is made available under a CC-BY 4.0 International license.

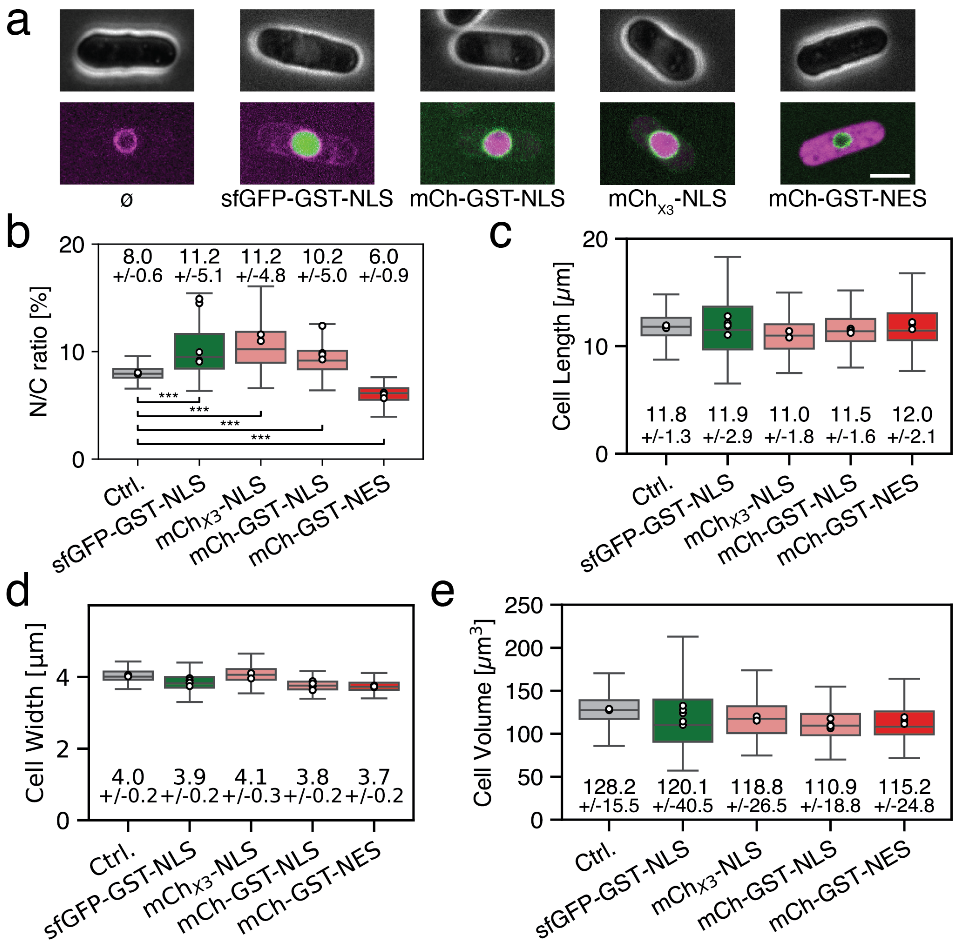

Supplementary Fig. 4 | Expression of other proteins besides mCherry-GST also alter the N/C ratio. **a,** Phase contrast images (top row) and mid-plane confocal images (bottom row) of representative cells for each plasmid construct shown in **Supplementary Fig. 1,b**, with the nuclear membrane and the exogenous protein tagged. **b,** Quantification of N/C ratios for populations of cells corresponding to the constructs in **a**, showing that neither mCherry nor GST alone drives changes in nuclear volume. Boxplots show single-cell N/C ratios, with each point representing the mean of an individual biological replicate. Statistical significance relative to the control was assessed using a linear mixed-effects model with strain as a fixed effect and replicate as a random intercept. Significance is indicated below each comparison (N≥162 cells per conditions). **c-e)** Quantification of the cell's geometrical parameters: cell length **e**, width **f**, and volume **g**. Mean values and standard deviation shown. Scale bar, 5 µm

bioRxiv preprint doi: https://doi.org/10.64898/2026.07.21.739918; this version posted July 23, 2026. The copyright holder for this preprint (which was not certified by peer review) is the author/funder, who has granted bioRxiv a license to display the preprint in perpetuity. It is made available under a CC-BY 4.0 International license.

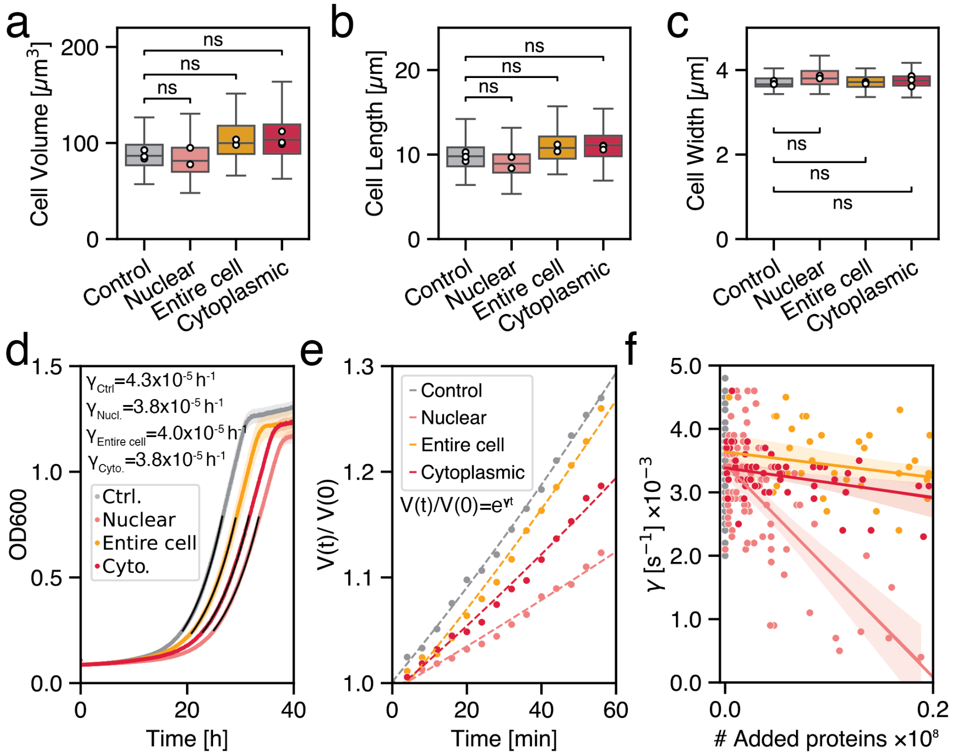

Supplementary Fig. 5 | Effects of expression of mCherry-based proteins on cell size and growth rate depend on their localization. a-c, Cells width, length and volumes measured using our pipeline for each condition described in Fig.1a. Statistical analysis was performed on replicate means using a one-way, followed by a Welch's t-test comparing each strain to the control strain (Control) with Bonferroni correction. **d,** Mean growth curves of cells in liquid culture for each condition (N=7 curves per conditions). Optical density at 600 nm (OD600) was measured over time. Population growth rates were estimated by fitting the exponential phase of the curves (black lines). **E,** Representative single-cell normalized volume trajectories over time for each strain. Each single-cell growth curve was fitted with a simple exponential function to obtain a single-cell growth rate (γ). **f,** Single-cell growth rates plotted as a function of the amount of extra protein per cell, for all condition (N>106 cells per condition).

bioRxiv preprint doi: https://doi.org/10.64898/2026.07.21.739918; this version posted July 23, 2026. The copyright holder for this preprint (which was not certified by peer review) is the author/funder, who has granted bioRxiv a license to display the preprint in perpetuity. It is made available under a CC-BY 4.0 International license.

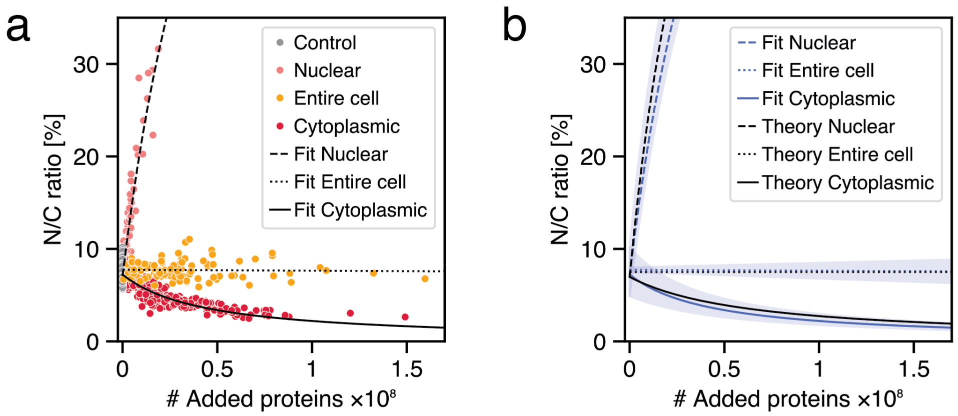

Supplementary Fig. 6 | Quantitative agreement between experimental data and theory strongly supports an osmotic model. a, Experimental data from Fig. 1d showing the N/C ratio as a function of the amount and subcellular localization of added exogenous protein. Fits to equations (7) and (8) provide estimates for the total protein quantity in the nucleus and the cell. **b,** Comparison between fit curves from **a** and theoretical curves, for each condition. Shaded regions in blue indicate the 1σ uncertainty of the fit curves in **a**. Theoretical curves (from Fig. 1d) are based upon a total protein number from proteomics data, which predict the expected behavior for the N/C ratio in each condition.

bioRxiv preprint doi: https://doi.org/10.64898/2026.07.21.739918; this version posted July 23, 2026. The copyright holder for this preprint (which was not certified by peer review) is the author/funder, who has granted bioRxiv a license to display the preprint in perpetuity. It is made available under a CC-BY 4.0 International license.

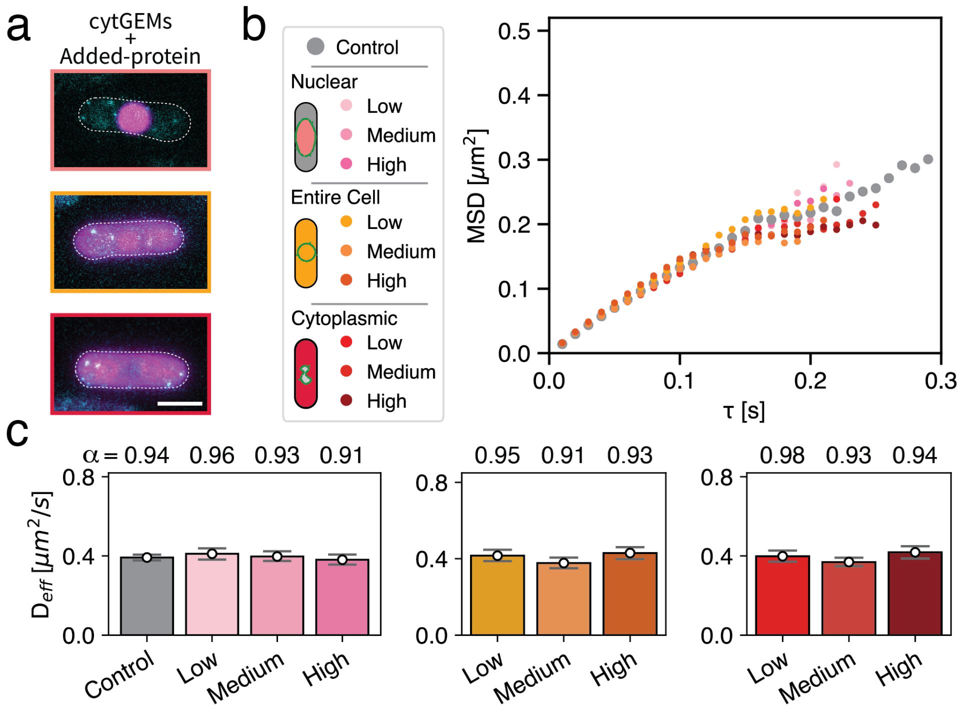

Supplementary Fig. 7 | Expression of mCherry-based proteins does not affect cytoplasmic mesoscale diffusivity. **a,** Representative fluorescence images of fission yeast expressing cytoplasmic GEMs (cyan) and the added protein tagged with mCherry (magenta). Scale bar 5 µm. **b,** Mean squared displacement (MSD) of cytoplasmic GEMs for each condition. Except for the control, the population was split into low, medium, and high levels of mCherry expression. **c,** Effective diffusion coefficient extracted from the GEM movements in each condition, plotted as a function of added protein expression level. Anomalous exponent (α) are indicated above each bar.

bioRxiv preprint doi: https://doi.org/10.64898/2026.07.21.739918; this version posted July 23, 2026. The copyright holder for this preprint (which was not certified by peer review) is the author/funder, who has granted bioRxiv a license to display the preprint in perpetuity. It is made available under a CC-BY 4.0 International license.

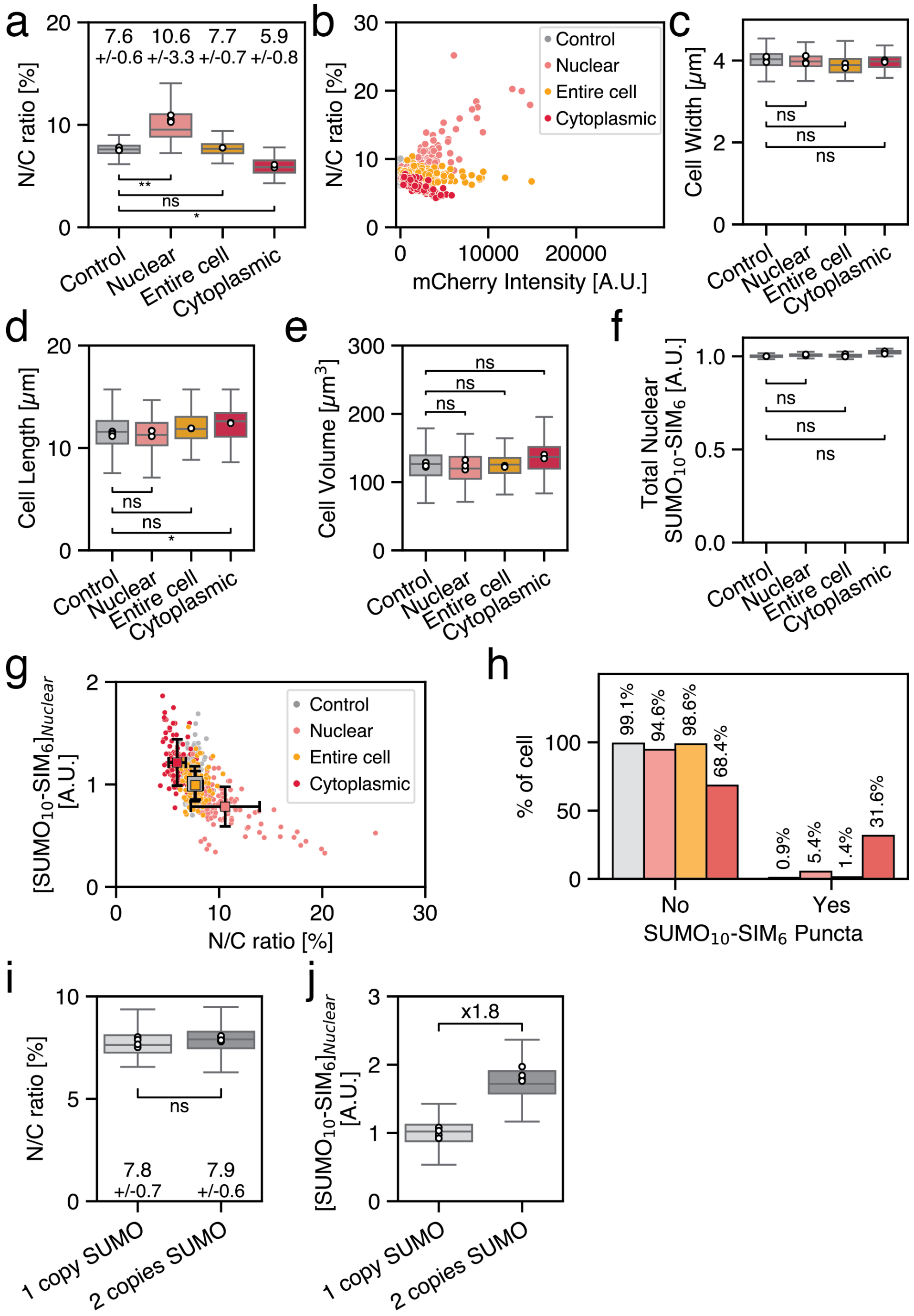

Supplementary Fig. 8 | Characterization of cells carrying one copy of the synthetic condensate gene. **a,** N/C ratio measured in populations of cells carrying one copy of the $SUMO_{10}$-SIM_6 synthetic condensate gene, expressing additional exogenous proteins (N≥98 cells per condition). Mean values and standard deviations are shown. **b,** N/C ratio plotted as a function of mCherry intensity for each condition in the one copy $SUMO_{10}$-SIM_6 background. **c-e,** Cells width, length and volume for cells carrying one copy of the $SUMO_{10}$-SIM_6, measured for each condition using an automated analysis pipeline. **f,** Total nuclear $SUMO_{10}$-SIM_6 concentration measured for each condition. **g,** Same as **f**, plotted for individual cells and mean values overlaid. **h,** Percentage of cells for which a nuclear $SUMO_{10}$-SIM_6 punctum was detected in each condition (N≥129 cells per condition). **i,** N/C ratio for populations of cells carrying one or two copies of the $SUMO_{10}$-SIM_6 construct, showing no difference in the N/C ratio between background. **j,** Nuclear concentration of $SUMO_{10}$-SIM_6 for the one- and two-copy strain backgrounds. Statistical analyses were performed on replicate means (white dots) using a one-way ANOVA, followed by pairwise Welch's t-test comparing each strain to the control.

bioRxiv preprint doi: https://doi.org/10.64898/2026.07.21.739918; this version posted July 23, 2026. The copyright holder for this preprint (which was not certified by peer review) is the author/funder, who has granted bioRxiv a license to display the preprint in perpetuity. It is made available under a CC-BY 4.0 International license.

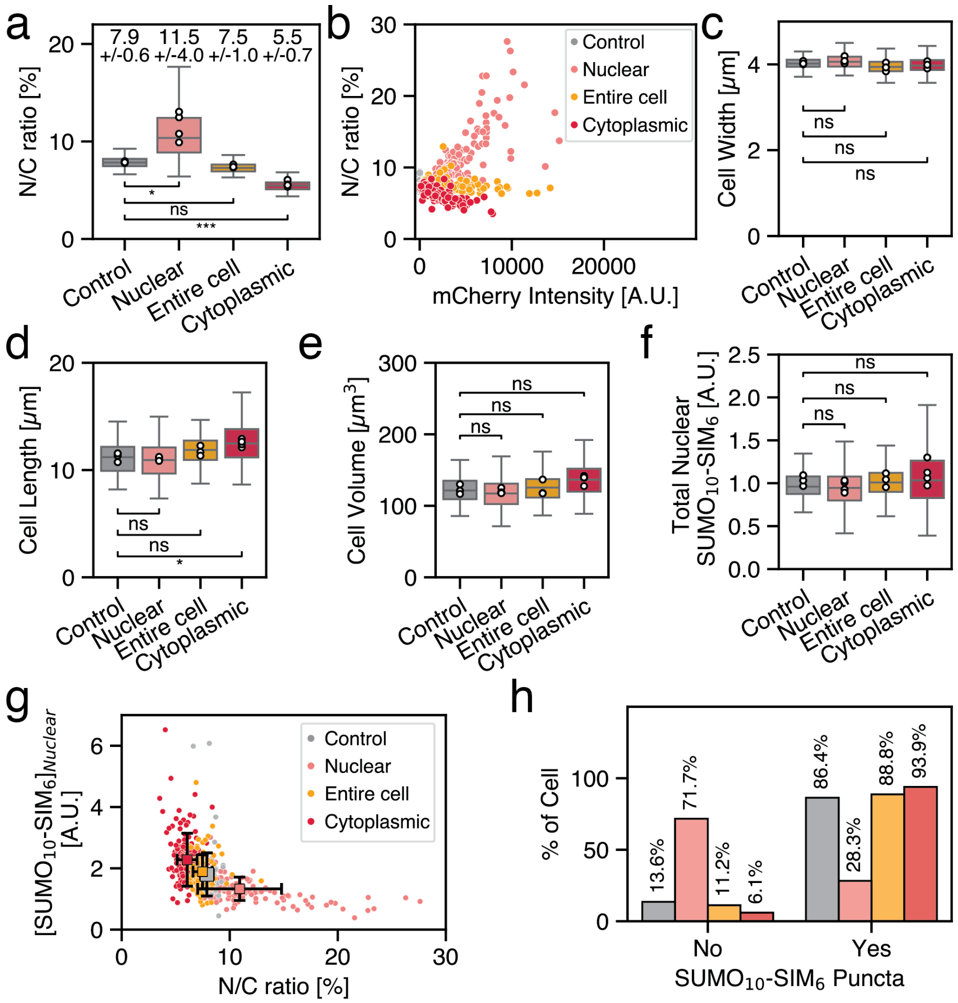

Supplementary Fig. 9 | Characterization of cells carrying two copies of the synthetic condensate gene. **a,** N/C ratio measured in populations of cells carrying two copies of the $SUMO_{10}$-SIM_6 synthetic condensate gene, expressing additional exogenous proteins (N≥107 cells per condition). Mean values and standard deviations are shown. **b,** N/C ratio plotted as a function of mCherry intensity for each condition in the two-copy $SUMO_{10}$-SIM_6 background. **c-e,** Cells width, length and volume for cells carrying two copies of the $SUMO_{10}$-SIM_6, measured for each condition using an automated analysis pipeline. **f,** Total nuclear $SUMO_{10}$-SIM_6 concentration measured for each condition. **g,** Same as **f**, normalized by the intensity of the 1 copy $SUMO_{10}$-SIM_6 background and plotted for individual cells and mean values overlaid. **h,** Percentage of cells for which a nuclear $SUMO_{10}$-SIM_6 punctum was detected in each condition (N≥103 cells per condition). Statistical analyses were performed on replicate means (white dots) using a one-way ANOVA, followed by pairwise Welch's t-test comparing each strain to the control.

bioRxiv preprint doi: https://doi.org/10.64898/2026.07.21.739918; this version posted July 23, 2026. The copyright holder for this preprint (which was not certified by peer review) is the author/funder, who has granted bioRxiv a license to display the preprint in perpetuity. It is made available under a CC-BY 4.0 International license.

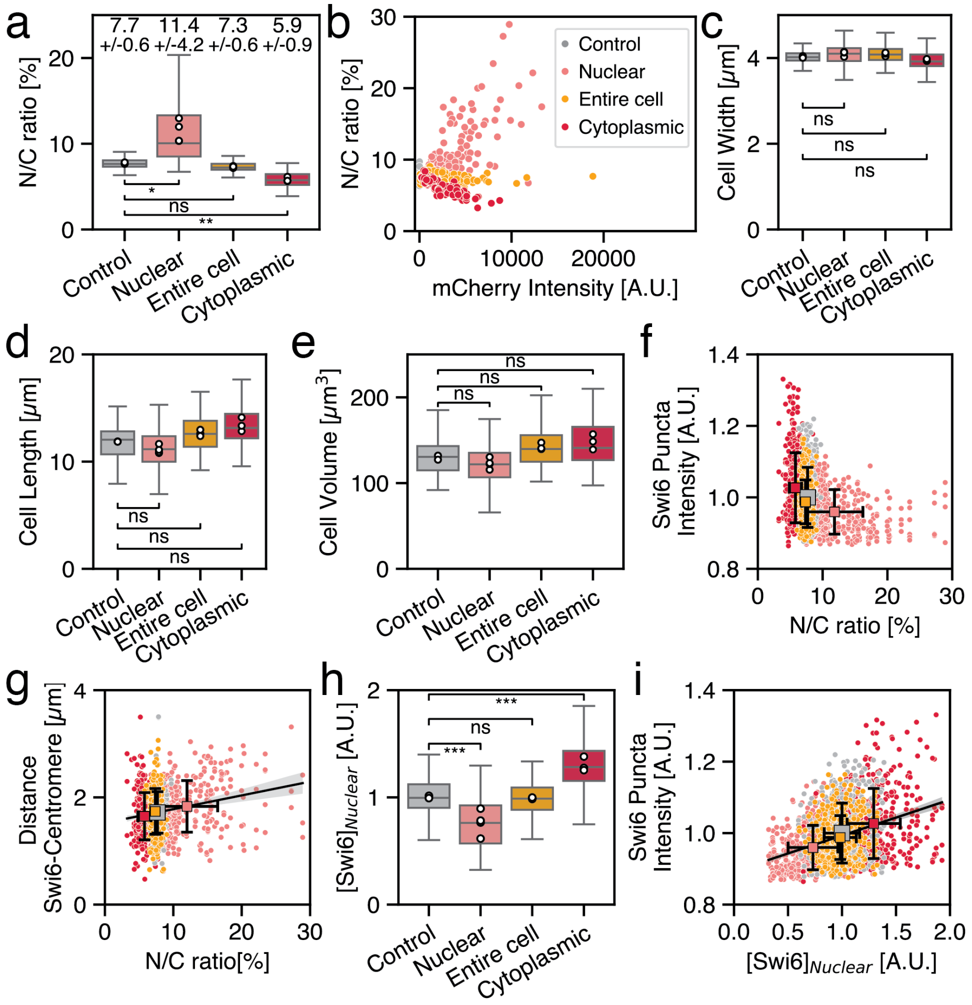

Supplementary Fig. 10 | Effect of nuclear size on Swi6-GFP puncta intensity and nuclear concentration. **a,** N/C ratio measured in populations of cells expressing additional exogenous proteins and carrying Swi6-tagged in their background (N≥188 cells per condition). Mean values and standard deviations are shown. **b,** N/C ratio plotted as a function of mCherry intensity for each condition. **c-e)** Cells width, length and volume measured for each condition. **f,** Swi6-GFP puncta intensity as a function of the N/C ratio, with means and standard deviation overlaid (N=1741 puncta). **g,** Distance between Swi6-GFP non-centromere puncta and the centromere as a function of the N/C ratio (black line indicates a linear fit with a positive slope; N=1741 puncta). **h,** Nuclear concentration of Swi6-GFP for each condition. **i,** Swi6-GFP puncta intensity plotted as a function of the nuclear concentration of Swi6-GFP. Means and standard deviation are overlaid. Statistical analyses were performed on replicate means (white dots) using one-way ANOVA tests (a,c-e,h).

bioRxiv preprint doi: https://doi.org/10.64898/2026.07.21.739918; this version posted July 23, 2026. The copyright holder for this preprint (which was not certified by peer review) is the author/funder, who has granted bioRxiv a license to display the preprint in perpetuity. It is made available under a CC-BY 4.0 International license.

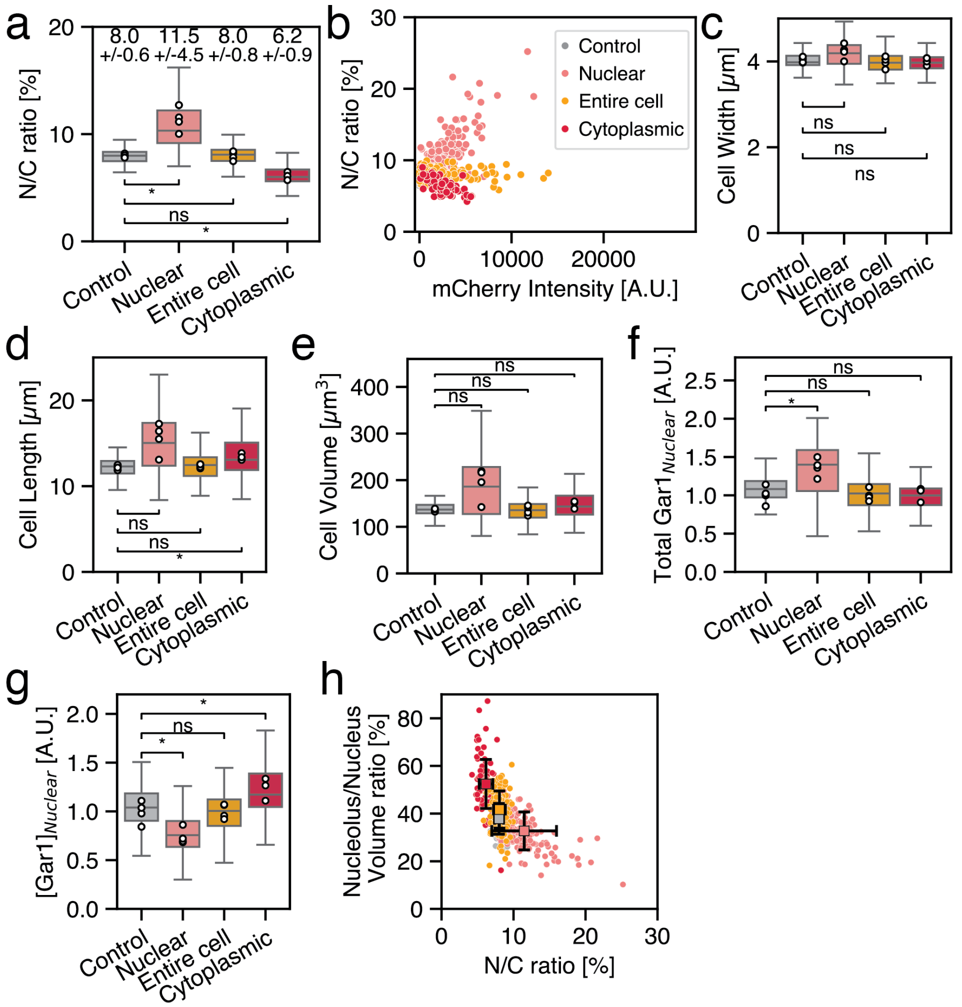

Supplementary Fig. 11 | Effects of nuclear size on the nucleolar marker Gar1-GFP. **a,** N/C ratio measured in populations of cells expressing additional exogenous proteins and carrying endogenously tagged Gar1 (N≥97 cells per condition). Mean values and standard deviations are shown. **b,** N/C ratio as a function of mCherry fluorescence intensity for each condition. **c-e)** Cells width, length and volume measured for each condition. **f,** Total nuclear Gar1-GFP signal for each condition **g,** Nuclear concentration of Gar1-GFP for each condition. **h,** Nucleolus to nucleus volume ratio as a function of the N/C ratio plotted per cell, with means and standard deviation overlaid for each condition. Statistical analyses were performed on replicate means (white dots) using one-way ANOVA (a,c-g).
